# Microbiota-based Signature of Gingivitis Treatments: A Randomized Study

**DOI:** 10.1038/srep24705

**Published:** 2016-04-20

**Authors:** Shi Huang, Zhen Li, Tao He, Cunpei Bo, Jinlan Chang, Lin Li, Yanyan He, Jiquan Liu, Duane Charbonneau, Rui Li, Jian Xu

**Affiliations:** 1 Single-Cell Center and Shandong Key Laboratory of Energy Genetics, Qingdao Institute of Bioenergy and Bioprocess Technology, Chinese Academy of Sciences, Qingdao, Shandong, 266101, China; 2 Department of Stomatology, Peking Union Medical College Hospital, Beijing, 100730, China; 3 Procter & Gamble Mason Business Center, Cincinnati, OH 45040, USA; 4 Procter & Gamble Innovation Center, Beijing 101312, China; 5 Procter & Gamble International Operations SA SG Branch, 138547, Singapore; 6 Sino-Danish Center for Education and Research, Beijing, 100190, China; 7 University of Chinese Academy of Sciences, Beijing, 100190, China

## Abstract

Plaque-induced gingivitis can be alleviated by various treatment regimens. To probe the impacts of various anti-gingivitis treatments on plaque microflora, here a double blinded, randomized controlled trial of 91 adults with moderate gingivitis was designed with two anti-gingivitis regimens: the brush-alone treatment and the brush-plus-rinse treatment. In the later group, more reduction in both Plaque Index (TMQHI) and Gingival Index (mean MGI) at Day 3, Day 11 and Day 27 was evident, and more dramatic changes were found between baseline and other time points for both supragingival plaque microbiota structure and salivary metabonomic profiles. A comparison of plaque microbiota changes was also performed between these two treatments and a third dataset where 50 subjects received regimen of dental scaling. Only *Actinobaculum*, TM7 and *Leptotrichia* were consistently reduced by all the three treatments, whereas the different microbial signatures of the three treatments during gingivitis relieve indicate distinct mechanisms of action. Our study suggests that microbiota based signatures can serve as a valuable approach for understanding and potentially comparing the modes of action for clinical treatments and oral-care products in the future.

Gingivitis is characterized by a tendency to bleed upon probing, and by changes in the color and texture of the gingivae^1^. Chronic gingivitis can progress to irreversible periodontitis, eventually leading to tooth loss^2^,^3^,^4^. Moreover, a low level of bacterial-induced gingival inflammation can induce a systemic increase in inflammatory markers^5^,^6^. Thus, the prevention and treatment of gingivitis are of particular clinical significance.

Dental plaque accumulation and changes in its microbial composition are together considered as two of the main causes of gingivitis^7^,^8^,^9^, therefore oral hygiene practices are used to control plaque and prevent/treat gingivitis. In addition, antimicrobial ingredients (such as stannous fluoride, cetylpyridinium chloride (CPC) and triclosan)^10^,^11^, which are typically effective against a wide range of bacteria, are frequently included in dentifrice and rinse formulations to improve their anti-plaque and anti-gingivitis efficacy^12^,^13^,^14^. For example, stannous fluoride gel has been shown to result in more than a 99% reduction in subgingival microbiota within 30 minutes in periodontal pockets^15^. Conversely, microbial sensitivity to antibacterial ingredients can vary significantly: *Enterococcus faecalis* (previously *Streptococcus faecalis*) and *Staphylococcus aureus* have been found to be more resistant to stannous fluoride than *Prevotella intermedia*^16^.

Despite the specific knowledge on interaction between individual plaque microbes and treatments, microbiota-wide understanding of the impacts of different anti-gingivitis treatments is limited. Different oral hygiene practices and antibacterial agents may have distinguishable impacts on plaque microbiota, which could generate distinct “microbial signatures” for the treatment regimens. We hypothesized that such distinct responses or signatures can be correlated with, or may even underlie, the development and reversal of gingivitis. The current randomized examiner-blinded study was designed to examine and compare the changes of oral microbiota during two treatment approaches: brush-plus-rinse and brush-alone. Between Baseline and Day 27, pyrosequencing of supragingival plaque and metabonomic analysis of saliva samples using Nuclear Magnetic Resonance (NMR) were performed to test bacterial correlations with gingival health. Additionally, a comparison was made between the plaque microbiota results (for brush-plus-rinse and brush-alone) and those from a separate study in which dental scaling was performed, so as to identify the microbial signatures of different treatments.

## Results

In total, 91 subjects completed the whole study. Five (2 from the brush-plus-rinse group and 3 from the brush-alone group) subjects dropped the study. Ten subjects missed at least one sampling for dental plaque or saliva (4 from the brush-plus-rinse group and 6 from the brush-alone group) during the study. No adverse events were reported or observed during the course of the study. Baseline demographic data were compared for the remaining 91 subjects. No significant difference was observed for age, gender, gingival index and plaque between two treatment groups at the Baseline (Table 1).

### Change of plaque amount and gingivitis symptom during the treatments

At Baseline, the TMQHI for the brush-plus-rinse group (n = 47) ranged from 2.78–3.99 while the mean MGI ranged from 1.40-2.44. For the brush-alone group (n = 44), the corresponding TMQHI and MGI values were 2.73–3.89 and 1.40–1.94, respectively. No statistically significant differences were observed between the groups (Table 1).

Starting from Day 3, both groups demonstrated statistically significantly lower TMQHI and mean MGI scores compared to Baseline (*p* < 0.001, two-side paired *t*-test) (Table 2). The brush-plus-rinse group also exhibited 5.08%, 14.1% and 20.2% greater mean MGI improvements than the brush-alone group for Days 3, 11 and 27 respectively (*p* < 0.05, ANCOVA), suggesting greater anti-gingivitis efficacy for the brush-plus-rinse treatment (Table 2). The brush-plus-rinse group also exhibited a 27.2%, 47.3% and 45.4% greater TMQHI improvement than the brush-alone group for Days 3, 11 and 27 respectively (*p < *0.0001, ANCOVA) (Table 2). No significant decrease in TMQHI was observed between Days 11 and 27 for either group (*p* > 0.05), suggesting that anti-plaque efficacy peaked for both at Day 11.

### Composition of plaque microbiota was profoundly altered during the treatments

**Figure 1:**
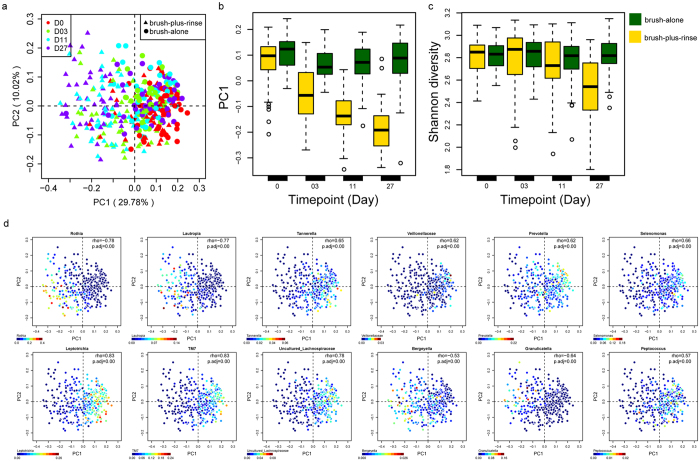
Dynamic changes in plaque microbiota. (**a**) Distinction in composition of the plaque microbiota at Baseline and following the treatments. All samples were plotted on the first two principal coordinates of the genus profile. (**b**) The PC1 value of each subject’s plaque microbiota (β diversity, Jensen–Shannon distance) significantly decreased for Treatment A. (**c**) The α diversity (Shannon diversity index) decreased significantly at Day 27 for Treatment A. (**d**) The 12 driver genera are displayed in blue (low abundance) and red (high abundance) in PCoA Plots.

To identify changes of plaque microbiota structure after product treatments in the study, all 364 microbiota from 91 subjects at four time points were clustered via principal coordinate analysis (PCoA) based on the relative abundance of genus-level taxa (Fig. 1a). Principal coordinate 1 (PC1) explained the largest amount of variation and was strongly associated with improvements in gingival health. The projected coordinate of a given microbiota on the first PC1 appeared to capture the gradient-like heterogeneity and compositional changes in plaque microbiota (Fig. 1b). The plaque microbiota in the brush-plus-rinse group exhibited more profound changes than brush-alone group during the study period inferred via significant changes of PC1 value (Fig. 1b). To identify changes of microbial diversity in the study, Shannon Index was calculated for each sample. A significant decrease in α diversity between Days 11 and 27 was observed only for the brush-plus-rinse group (Fig. 1c). This change was driven in part by lower detection rate of some bacterial taxa after treatments (Fig. 1d).

For the 91 subjects considered at all four time points, 12 bacterial genera were found to be the drivers of microbiota heterogeneity along PC1 including: *Rothia*, *Bergeyella*, *Lautropia*, *Granulicatella*, *Prevotella, Leptotrichia*, *Selenomonas*, uncultured *Lachnospiraceae*, TM7, *Tannerella*, *Peptococcus*, and unclassified *Veillonellaceae*. Four of these – *Rothia*, *Granulicatella*, *Bergeyella* and *Lautropia* – decreased in relative abundance (“positive drivers”), while eight increased (“negative drivers”) (Fig. 1d). Their gradients in abundance were significantly correlated with the coordinates of their corresponding samples on PC1 (Spearman *rho* > 0.5, FDR *q* < 0.2). Interestingly, relative abundance changes for *Leptotrichia, Rothia, TM7 genera* and *Lautropia* of each subject from Baseline to Day 11 were also significantly correlated with improvements in gingival health (mean MGI changes) from Baseline to Day 27 (Fig. S1). Thus, these bacterial biomarkers might be useful for evaluating the anti-gingivitis efficacy of products over a relatively short time period.

### Saliva metabolic profiles were altered after product usage

**Figure 2:**
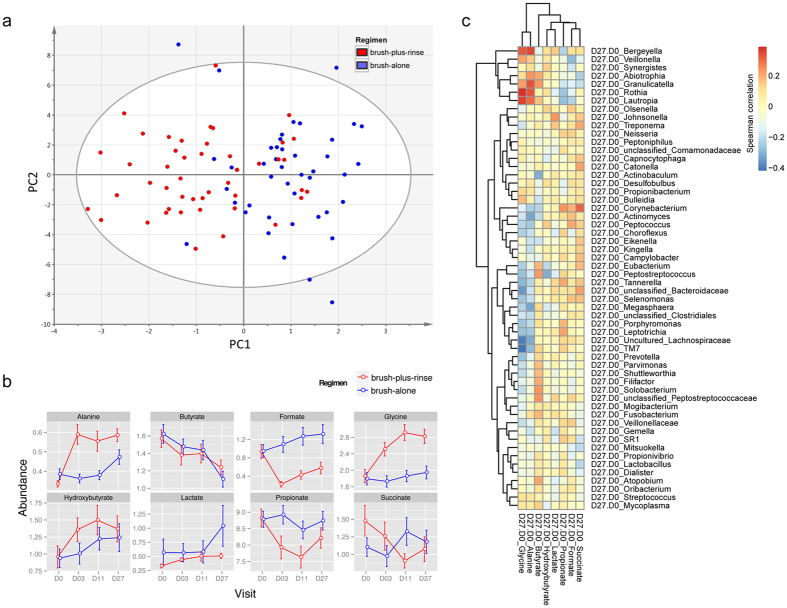
Metabonomic analysis of saliva samples. (**a**) OPLS analysis of NMR data revealed the metabolite changes from Baseline to Day 27 for each subject: the brush-plus-rinse and the brush-alone groups are marked in red and blue respectively. (**b**) Changes of eight typical metabolites along the full duration of study were respectively compared between the two treatment groups. Arbitrary unit for each metabolite was calculated from normalized NMR spectrum. (**c**) The heatmap indicates correlations between metabolite and plaque microbiota changes. Eight metabolites in saliva were calculated and normalized. Spearman correlation for metabolite changes and oral bacteria was calculated (Baseline to Day 27). The R-value is shown in blue (low) and red (high). Different bacteria and metabolites were clustered by their relative abundance changes after the respective 27-day treatments.

Saliva has been extensively examined in attempts to assess the oral disease status^17^. In this study, metabonomic analysis revealed significant difference in metabolite profiles between the brush-plus-rinse group and the brush-alone group at Day 27 (Fig. 2a). The prominent metabolites in saliva were compared across the study. For both brush-plus-rinse group and brush-alone group, abundance of butyrate was decreased, while hydroxybutyrate and lactate were increased from Day 0–Day 27. For the brush-plus-rinse group, the abundance of propionate, formate, and succinate decreased in comparison to the brush-alone group, while alanine and glycine increased (Fig. 2b). The metabolite changes in saliva were also correlated with the changes of microbial composition and quality in plaque (Fig. 2c). For example, propionate was positively correlated with *Tanneralla*, which was significantly reduced in plaque microbiota during the product treatments (Figs 1d and 2c). Propionate is a virulence factor produced by *T. forsythia* for immunoevasion and immunosuppression^18^,^19^. Alanine and glycine were used for oral bacterial cell wall-peptidoglycan synthesis^20^,^21^. Increased level of alanine and glycine may result from reduced total oral bacterial load and decreased cell wall synthesis activity from chemostatic effects of product treatments.

### Microbial signatures of the brush-plus-rinse, brush-alone and dental scaling treatments

**Figure 3:**
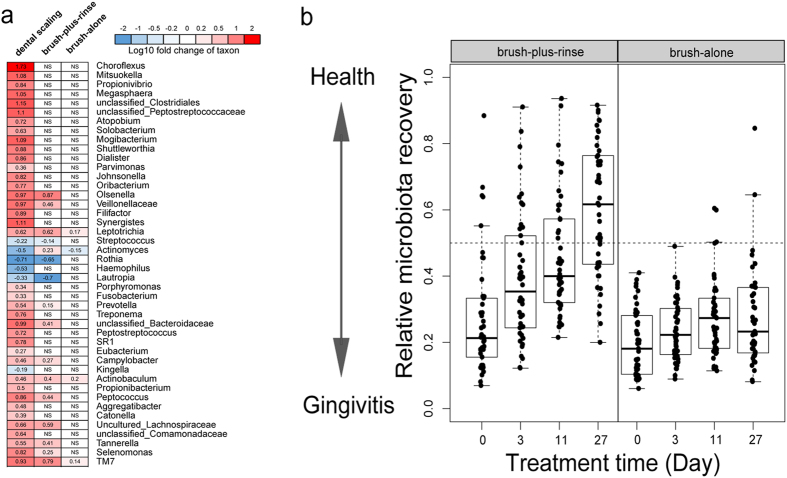
Microbial signature of different anti-gingivitis treatments and evaluation of the relative microbiota recovery for the two treatment groups. (**a**) The heat map showed the enrichment of each bacterial genus from the disease state to the health state during the various treatments. During dental scaling, 44 bacterial genera significantly changed, representing the most extensive microbiota change among the three treatments. Therefore, the change pattern of dental scaling is used as a reference to evaluate the other two treatments. In certain cells, “NS” is displayed, which indicates the change (before and after a certain treatment) of those particular genera was “Not Significant”. (**b**) Use of plaque-microbiota-based model of dental scaling to stratify subjects in the brush-plus-rinse group and the brush-alone group at four consecutive time points. Boxes denote the IQR between the first and third quartiles, and the line within denotes the median; whiskers denote the lowest and highest values within 1.5 times the IQR.

Dental scaling is widely considered as the most effective anti-gingivitis treatment. A historical published 16S rDNA pyrosequencing data of dental scaling was used for identifying its specific microbial signature during gingivitis regression^8^. Totally 44 taxa changed significantly (corrected *p* < 0.01, Wilcoxon signed rank test; Fig. 3a). By comparing the microbial signatures across the three treatments, we found that only *Actinobaculum*, TM7 and *Leptotrichia* significantly decreased during gingivitis regression. Except for *Actinomyces*, relative abundance changes of 15 taxa after the brush-plus-rinse treatment were in the same direction (increase or decrease) as the dental scaling group (Fig. 3a). For the brush-alone treatment, three oral taxa (*Actinobaculum*, TM7 and *Leptotrichia*) were significantly reduced, while only *Actinomyces* was significantly increased (Fig. 3a). Thus, different anti-gingivitis treatments generate distinct microbial signatures, which may link to clinical symptoms.

To explore the potential of applying dental-scaling-associated microbiota shifts as a reference to assess the various anti-gingivitis treatments, we trained a random forests model via the training set of 50 subjects with both naturally-occurred gingivitis (i.e. NG) and post-dental-scaling healthy status using the genus profiles. The discriminatory power of the Random Forests model derived from the dental scaling dataset was calculated as the area under the ROC curve (AUC 0.99) (Fig. S2A). The lowest error rate for cross-validation in training data was obtained when including taxa beyond the top five (Fig. S2B). Therefore, this Random Forests model can be used to define relative microbiota changes for subjects receiving a given anti-gingivitis treatment. To test the extent to which this could be applied, we stratified the brush-plus-rinse and the brush-alone groups at four consecutive time points using this model (Fig. 3b). The median relative microbiota recovery of the brush-plus-rinse subjects with gingivitis increased linearly over time and eventually reached 60% at Day 27, while few of the brush-alone subjects experienced changes in plaque microbiota or a shift to “health”. Thus the pattern of microbiota responses was highly correlated with reductions in gingivitis. Furthermore, the results validate a compact version of this predictive model that includes the 15 most discriminatory taxa as identified in the MiG15 model previously reported^8^. Such relative microbiota recovery, defined by either all of the important taxa or only the top 15, can readily distinguish anti-gingivitis efficacy (Fig. 3b, S3).

## Discussion

Our data here provide the first landscape view on how oral care products impact plaque microbiota and associated metabolic activities *in vivo*. Firstly, our results revealed that treatments lead to distinct temporal pattern of microbial composition changes during the reversal of gingivitis (Fig. 1 and Table 2). Intriguingly, brush-plus-rinse group exhibited more profound temporal changes in both α and β diversity of plaque microbiota than brush-alone group (Fig. 1b,c). Furthermore, by comparing the brush-plus-rinse and the brush-alone groups with a separate cohort of subjects that received dental scaling, all treatments were stratified by plaque microbiota responses. Remarkably, a treatment-specific microbiota signature was identified, suggesting distinct antimicrobial mechanisms between different anti-gingivitis treatments (Fig. 3a).

Secondly, these results also provided a basis for understanding the structural and functional consequences of oral microbiota as altered by a certain gingivitis treatment. For dental scaling, which is one of the most effective periodontal therapies, Socransky *et al.* reported that periodontal pathogens including *Tannerella forsythia*, *Treponema denticola* and *Eubacterium nodatum* were significantly reduced in pockets of periodontitis^22^. In those receiving dental scaling in our study, *Tannerella*, *Treponema* and *Eubacterium* were also reduced (in the supragingival plaque), adding more evidence to the microbial link between periodontitis and gingivitis. During the brush-plus-rinse treatment with oral care products containing sodium fluoride/stannous chloride and CPC, twelve bacteria were significantly affected, ten of which behaved consistently during dental scaling^8^: *Rothia* and *Lautropia* were more abundant in plaque microbiota in gingival health, while *Prevotella, Leptotrichia*, *Selenomonas*, uncultured *Lachnospiraceae*, TM7, *Tannerella*, *Peptococcus* and unclassified *Veillonellaceae* were more abundant with gingivitis. The brush-plus-rinse treatment increased relative abundance of two oral bacteria associated with gingival health and decreased that of eight associated with gingivitis in supragingival plaque. Two factors may have contributed to the anti-microbial effects of the brush-plus-rinse treatment: (*i*) reactive oxygen species (ROS) generated by stannous chloride and (*ii*) CPC (as a cationic surfactant), both of which selectively inhibited growth of obligate anaerobes (including *Prevotella, Leptotrichia*, *Selenomonas*, *Tannerella* and *Peptococcus*)^23^,^24^,^25^.

Thirdly, our data strongly argue for the importance of unclassified and uncultured bacterial phyla in gingivitis development and treatment. For example, unclassified Clostridiales, unclassified Peptostreptococcaceae, unclassified Bacteroidaceae, SR1, uncultured Lachnospiraceae, unclassified Comamonadaceae and TM7 were detected in oral microbiome and were significantly reduced after dental scaling. Interestingly, TM7 was reduced across the three different treatments, which was recently found to lead a unique parasitic lifestyle and could modulate host immune response *in vitro*^26^.

Fourthly, salivary metabolic profiles were also significantly altered, in parallel with the shift in plaque microbiota and gum health improvement. This is consistent with a previous report in which the level of salivary propionate and succinate was greater in chronic periodontitis subjects than in subjects with healthy gingivae^27^. Of significance, short chain fatty acids such as acetate, propionate, n-butyrate and succinate also regulate human gingival epithelial cell proliferation and keratin expression, and modulate leukocyte response^28^,^29^.

Recently multiple reports have suggested that the composition of plaque microbiota can be linked to the state of periodontal diseases^8^,^30^,^31^,^32^,^33^. Our results suggest that microbiome shifts, depicted as microbial signatures, could be applied for comparing the mechanisms of different oral care products or treatments and also for predicting their clinical efficacy. Future research with different population groups, different time periods and larger cohorts, should further test the general applicability of our approach. In addition, the treatment-discriminatory taxa, identified by the Random Forests model here, may themselves be therapeutic candidates and/or form the basis for high-throughput, low-cost assessments of oral-care products and regimens.

## Methods

### Subject cohort

The study (Clinicaltrials.gov NCT02401360; registration date: March 24, 2015) was conducted at Procter & Gamble (Beijing) Technology Co., Ltd. Oral Care Department, with approval from the P&G Beijing Technical Center (China) Institutional Review Board and in accordance with the ICH Guidelines for Good Clinical Practice. A total of 106 subjects with gingivitis were enrolled, after signing an informed consent form. Subjects were recruited between March 16 and March 31 in 2013, and follow-up dates were from April 04–April 30. The planned sample size of 100 subjects (50 subjects per group) will provide at least 80% power to detect a mean difference between treatment groups of at least 0.153 in mean Mazza Gingival Index (MGI) using two-sided testing at a 5% significance level. This estimate assumes the standard deviation for mean MGI scores is 0.27 or smaller. Individuals who met the following criteria were included into the study: be at least 18 years of age; have a minimum of 18 natural teeth with facial and lingual scorable surfaces; have at least 10 bleeding sites; mean MGI is from 1.0–2.5 at Baseline; be in good general health as determined by the Investigator/designee based on a review of the medical history/update for participation in the study. Exclusion criteria include: severe periodontal disease, as characterized by presence of four or more teeth with ≥5 mm pockets in two quadrants , purulent exudates, generalized mobility, and/or severe recession; any condition that requires antibiotic premedication for the administration of a dental prophylaxis; self-reported pregnancy or intent to become pregnant during the course of the study and nursing females; fixed facial orthodontic appliances; atypical discoloration or pigmentation in the gingival tissue; chronic diseases including hepatitis, diabetes, or other communicable diseases and conditions that require prophylactic antibiotic coverage prior to invasive procedures; any diseases or conditions that could be expected to interfere with the subject safely completing the study. Smoking status was recorded for all the recruited subjects. Only one subject (male, 27 years old) in the brushing-plus-rinsing group had smoking history. To minimize the impact of smoking on gingivitis development/recovery in this study, all subjects were refrained from smoking across this study.

The threshold used to define periodontitis varies among different studies^34^. Querna *et al.* used a threshold of pocket probing depth (PPD) of ≥5 mm to define moderate to advanced periodontitis^35^. As our study cohort was designed to include those subjects with gingival bleeding but not with periodontitis, subjects with four or more teeth with ≥5 mm pockets in two quadrants were excluded from our study. Moreover, gingival bleeding was assessed by Mazza Gingival Index, while change of pocket depth was not evaluated.

### Treatment protocols

This is an examiner-blind, single-center, two-leg and 27-day clinical study. At the baseline visit, enrolled subjects were stratified based on gender, age, the average number of bleeding sites and smoking status. Within the strata, subjects were randomly assigned to one of the treatment groups. Subjects residing in the same household were assigned to the same treatment group. The brush-plus-rinse group (n = 53) was instructed to brush twice-daily with a CrossAction manual toothbrush (Crest) and a toothpaste containing 0.321% sodium fluoride and 1.16% stannous chloride (Crest). The brush-plus-rinse group was also instructed to use 20 ml rinse with 0.0747% CPC (Crest) for 30 seconds after brushing. The brush-alone group (n = 53) was instructed to brush twice-daily with a manual brush (Crest) and a 0.243% sodium fluoride toothpaste (Crest). During the course of the trial, the entire cohort visited clinical site for four times. Clinical trial manager would ensure compliance with the product usage. Adverse events will be collected at each visit during the study. There were no adverse events reported in this study, and both treatments were well-tolerated.

### Clinical assessment and statistical analysis

Plaque was scored on the six surfaces of all teeth (excluding 3rd molars, crowns and surfaces with cervical restorations) in accordance with the Turesky Modification of the Quigley-Hein Index (TMQHI)^36^. Gingivitis was assessed using the Mazza Gingival Index (MGI)^8^. To investigate examiner repeatability and reliability with respect to gingivitis, separate studies were performed for examiner qualification. The examiner could detect the difference between prophylaxis and non-prophylaxis consistently. Baseline demographic data were compared between the brush-plus-rinse and brush-alone groups (ANOVA for age and a Two-sided Fisher’s exact test for gender). Treatment comparisons were analyzed using analysis of covariance models with corresponding baseline as the covariate (ANCOVA).

### Plaque sampling and analysis

Two entire quadrants (1&3 or 2&4) of supragingival plaque samples (along the gumline within 2 mm depth) were collected by dentists using a Gracey curette at each visit after clinical assessments^8^. Total DNA of plaque samples were extracted and PCR amplicon libraries of 16S rRNA gene V1-V3 hyper-variable region (*Escherichia coli* positions 5–534) were pyrosequenced according to our published protocols^37^,^38^.

The sequencing data was analyzed with MOTHUR^39^, which yielded a total of 1,232,560 processed reads (average 2988 reads per sample; range 639–33805). Sequences were assigned to operational taxonomic units (OTUs) with a 97% threshold of pairwise identity, then classified using the Oral CORE reference database (CI 80%)^40^. In addition, to eliminate any potential influence from uneven sampling, 639 sequences/sample were randomly selected and used to compare α and β diversity. The function “diversity” in the “vegan” package of R was used to calculate α diversity indices, and structural heterogeneity determined by clustering plaque microbiota using the principal coordinates analysis (PCoA) of the Jensen-Shannon divergence (JSD) matrix, based on the normalized abundance of genus. Genera with<0.1% average abundance across all samples had first been removed to decrease noise. The 454 sequence data were submitted to the NCBI Sequence Read Archive under **Accession ID SRP045295**.

A historical published 16S rDNA pyrosequencing data of dental scaling, which also originated from this team and followed identical pyrosequencing strategy and procedure^8^, was used for comparing microbial signature and predicting anti-gingivitis effects of different treatments. In that study, after receiving first dental prophylaxis (super and sub gingival prophylaxis) and tooth polishing, each subject was instructed to brush under supervision for three minutes with a marketed anti-cavity dentifrice and then use the floss to clean the dental interproximal area for three weeks. During this period, subjects could receive up to another two dental prophylaxes in order to reach optimal gum health status. These two studies used the same subject recruiting criteria and applied identical clinical sample collection, processing and sequencing protocols. We then trained a Random Forests model of 50 subjects with gingivitis, and reversal of gingivitis following dental scaling, using genus profiles^41^. Ten-fold cross-validation was used to estimate dental-scaling-discriminatory performance as a function of the number of top-ranking taxa.

### Metabonomic analysis

Saliva samples were thawed at 25 °C and centrifuged for 30 minutes to remove any insolubles. For each sample, an aliquot of 0.72 mL supernatant was transferred to a 5 mm NMR tube and was mixed with 80 μL PBS/D2O as reference solution and 80 μL pyridazine for chemical shifts normalization and calibration. The proton NMR spectra were collected on a 500 MHz Bruker AVIII NMR spectrometer equipped with a 5 mm liquid probe. A 1D-NOESY pulse sequence with pre-saturation water suppression was used. The proton NMR spectra were first calibrated by acetate peak at 1.95 ppm and the spectra segment was integrated. Before OPLS analysis using SIMCA-p 13 software (Umetrics), spectrum data for Days 3, 11 and 27 was subtracted by Baseline (Day 0) value. Key chemical shifts identified by OPLS analysis can be further linked to typical metabolites by comparing NMR spectrum of pure chemicals. Relative concentration for each metabolite was calculated by the peak integration in TOPSPIN v. 3.1 (Bruker). To test the links between identified salivary metabolites and the plaque microbiota taxa, Spearman correlation analysis was performed (CI 95%; *p* < 0.05).

## Additional Information

**How to cite this article**: Huang, S. *et al.* Microbiota-based Signature of Gingivitis Treatments: A Randomized Study. *Sci. Rep.* **6**, 24705; doi: 10.1038/srep24705 (2016).

## Supplementary Material

Supplementary Information

## Figures and Tables

**Table 1:** Demographics of the randomized subjects at the baseline.

Demographic/Statistic or Category	Brush-plus-rinse group	Brush-alone group	Overall	P-value
	(n = 47)	(n = 44)	(n = 91)	
Age (Years)				
Mean (SD)	34.70 (8.56)	32.84 (7.00)	33.80 (7.86)	0.2612^a^
Min.-Max.	18–50	20–50	18–50	
Gender				
Female^b^	42 (89%)	42 (95%)	84 (92%)	0.4362^c^
Male^b^	5 (11%)	2 (5%)	7 (8%)	
Gingival index (mean MGI)				
Mean (SD)	1.68 (0.203)	1.68 (0.157)	1.68 (0.181)	0.8855^a^
Min.-Max.	1.40 to 2.44	1.40 to 1.94	1.40 to 2.44	
Plaque Index (TMQHI)				
Mean (SD)	3.40 (0.298)	3.29 (0.274)	3.34 (0.290)	0.0768^a^
Min.-Max.	2.78 to 3.99	2.73 to 3.89	2.73 to 3.99	

^a^Two-sided ANOVA p-value for the treatment comparison.

^b^The number (percent) of subjects in each category.

^c^Two-sided Fisher’s exact test p-value for the treatment comparison.

**Table 2:** Comparisons of plaque index (TMQHI) and gingival index (mean MGI) between treatments.

Clinical assessments	Brush-plus-rinse group (n = 47)	Brush-alone group (n = 44)	Percentage of Diff vs. Brush-alone group^a^	P-value^b^
Plaque Index (TMQHI)-Adj. Mean (SE)				
Day 03	2.170 (0.045)	2.981 (0.047)	27.20%	<0.0001
Day 11^c^	1.566 (0.057)	2.969 (0.059)	47.30%	<0.0001
Day 27	1.672 (0.063)	3.061 (0.065)	45.40%	<0.0001
Gingival index (mean MGI)-Adj. Mean (SE)				
Day 03	1.514 (0.023)	1.595 (0.024)	5.08%	0.0158
Day 11^c^	1.347 (0.024)	1.568 (0.025)	14.10%	<0.0001
Day 27	1.156 (0.019)	1.450 (0.019)	20.20%	<0.0001

^a^Percent Difference = 100× ((Brush-alone group - Brush-plus-rinse group)/Brush-alone group)

^b^2-sided p-value comparing treatments using analysis of covariance (ANCOVA).

^c^One subject’s clinical data are missed at this timepoint for the brush-plus-rinse group.

## References

[b1] Armitage G. C. Periodontal diagnoses and classification of periodontal diseases. Periodontol. 2000 34, 9–21, doi: 10.1046/j.0906-6713.2002.003421.x (2004). 14717852

[b2] Sheiham A. Is the chemical prevention of gingivitis necessary to prevent severe periodontitis? *Periodontol*. 2000 15, 15–24, doi: 10.1111/j.1600-0757.1997.tb00100.x (1997). 9643228

[b3] Loesche W. Dental caries and periodontitis: contrasting two infections that have medical implications. Infect. Dis. Clin. North Am. 21, 471–502, vii, doi: 10.1016/j.idc.2007.03.006 (2007). 17561079

[b4] Ramseier C. A. *et al.* Identification of pathogen and host-response markers correlated with periodontal disease. J. Periodontol. 80, 436–446, doi: 10.1902/jop.2009.080480 (2009). 19254128 PMC5695217

[b5] Eberhard J. *et al.* Experimental gingivitis induces systemic inflammatory markers in young healthy individuals: a single-subject interventional study. PLoS One 8, e55265, doi: 10.1371/journal.pone.0055265 (2013). 23408963 PMC3567060

[b6] Ylostalo P. V., Jarvelin M. R., Laitinen J. & Knuuttila M. L. Gingivitis, dental caries and tooth loss: risk factors for cardiovascular diseases or indicators of elevated health risks. J. Clin. Periodontol. 33, 92–101, doi: 10.1111/j.1600-051X.2005.00875.x (2006). 16441731

[b7] Haraszthy V. I., Zambon J. J. & Sreenivasan P. K. The antimicrobial efficacy of commercial dentifrices. *Gen*. Dent. 58, 50–55;quiz 56–57, 79–80 (2010). 20129893

[b8] Huang S. *et al.* Predictive modeling of gingivitis severity and susceptibility via oral microbiota. ISME J 8, 1768–1780, doi: 10.1038/ismej.2014.32 (2014). 24646694 PMC4139724

[b9] Kistler J. O., Booth V., Bradshaw D. J. & Wade W. G. Bacterial community development in experimental gingivitis. PLoS One 8, e71227, doi: 10.1371/journal.pone.0071227 (2013). 23967169 PMC3743832

[b10] Boneta A. E. *et al.* Comparative investigation of the efficacy of triclosan/copolymer/sodium fluoride and stannous fluoride/sodium hexametaphosphate/zinc lactate dentifrices for the control of established supragingival plaque and gingivitis in a six-month clinical study. J. Clin. Dent. 21, 117–123 (2010). 21269041

[b11] Costa X. *et al.* Efficacy of a new mouth rinse formulation based on 0.07% cetylpyridinium chloride in the control of plaque and gingivitis: a 6-month randomized clinical trial. J. Clin. Periodontol. 40, 1007–1015, doi: 10.1111/jcpe.12158 (2013). 24024983

[b12] Cagetti M. G. *et al.* Effect of a toothpaste containing triclosan, cetylpyridinium chloride, and essential oils on gingival status in schoolchildren: a randomized clinical pilot study. Quintessence Int. 46, 437–445, doi: 10.3290/j.qi.a33530 (2015). 25646169

[b13] Cortelli S. C., Cortelli J. R., Shang H., Costa R. & Charles C. A. Gingival health benefits of essential-oil and cetylpyridinium chloride mouthrinses: a 6-month randomized clinical study. Am. J. Dent. 27, 119–126 (2014). 25208357

[b14] Hernandez-Cott P. L., Elias Boneta A., Stewart B., DeVizio W. & Proskin H. M. Clinical investigation of the efficacy of a commercial mouthrinse containing 0.05% cetylpyridinium chloride in reducing dental plaque. J. Clin. Dent. 20, 39–44 (2009). 19591335

[b15] Oosterwaal P. J., Mikx F. H., van ‘t Hof M. A. & Renggli H. H. Short-term bactericidal activity of chlorhexidine gel, stannous fluoride gel and amine fluoride gel tested in periodontal pockets. J. Clin. Periodontol. 18, 97–100 (1991). 2005233 10.1111/j.1600-051x.1991.tb01696.x

[b16] Meurman J. H. *et al.* Activity of amine-stannous fluoride combination and chlorhexidine against some aerobic and anaerobic oral bacteria. Oral Microbiol. Immunol. 4, 117–119, doi: 10.1111/j.1399-302X.1989.tb00109.x (1989). 2762015

[b17] Barnes V. M. *et al.* Metabolomics reveals elevated macromolecular degradation in periodontal disease. J. Dent. Res. 90, 1293–1297, doi: 10.1177/0022034511416240 (2011). 21856966

[b18] Takemoto T., Kurihara H. & Dahlen G. Characterization of Bacteroides forsythus isolates. J. Clin. Microbiol. 35, 1378–1381 (1997). 9163447 10.1128/jcm.35.6.1378-1381.1997 PMC229752

[b19] Sanghavi T. H., Shah N., Shah R. R. & Sanghavi A. Investigate the correlation between clinical sign and symptoms and the presence of P. gingivalis, T. denticola, and T. forsythia individually or as a “Red complex” by a multiplex PCR method. J Conserv Dent 17, 555–560, doi: 10.4103/0972-0707.144604 (2014). 25506144 PMC4252930

[b20] Fukuhara H., Umemoto T., Sagawa H., Kato K. & Kotani S. Purification and quantitative chemical analysis of cell wall peptidoglycans of Leptotrichia buccalis. Infect. Immun. 39, 132–136 (1983). 6822410 10.1128/iai.39.1.132-136.1983 PMC347914

[b21] Baboolal R. Cell wall analysis of oral filamentous bacteria. J. Gen. Microbiol. 58, 217–226 (1969). 5360478 10.1099/00221287-58-2-217

[b22] Socransky S. S. *et al.* Effect of periodontal therapy on the subgingival microbiota over a 2-year monitoring period. I. Overall effect and kinetics of change. J. Clin. Periodontol. 40, 771–780, doi: 10.1111/jcpe.12117 (2013). 23710672 PMC3757950

[b23] Cabiscol E., Tamarit J. & Ros J. Oxidative stress in bacteria and protein damage by reactive oxygen species. Int Microbiol 3, 3–8 (2000). 10963327

[b24] de Mattos J. C. *et al.* Damage induced by stannous chloride in plasmid DNA. Toxicol. Lett. 116, 159–163, doi: 10.1016/S0378-4274(00)00213-7 (2000). 10906433

[b25] Sreenivasan P. K., Haraszthy V. I. & Zambon J. J. Antimicrobial efficacy of 0.05% cetylpyridinium chloride mouthrinses. Lett. Appl. Microbiol. 56, 14–20, doi: 10.1111/lam.12008 (2013). 23039819

[b26] He X. *et al.* Cultivation of a human-associated TM7 phylotype reveals a reduced genome and epibiotic parasitic lifestyle. Proc. Natl. Acad. Sci. USA 112, 244–249, doi: 10.1073/pnas.1419038112 (2015). 25535390 PMC4291631

[b27] Aimetti M., Cacciatore S., Graziano A. & Tenori L. Metabonomic analysis of saliva reveals generalized chronic periodontitis signature. Metabolomics 8, 465–474, doi: 10.1007/s11306-011-0331-2 (2012).

[b28] Pollanen M. T. & Salonen J. I. Effect of short chain fatty acids on human gingival epithelial cell keratins *in vitro*. Eur. J. Oral Sci. 108, 523–529, doi: 10.1034/j.1600-0722.2000.00881.x (2000). 11153927

[b29] Vinolo M. A., Rodrigues H. G., Nachbar R. T. & Curi R. Regulation of inflammation by short chain fatty acids. Nutrients 3, 858–876, doi: 10.3390/nu3100858 (2011). 22254083 PMC3257741

[b30] Griffen A. L. *et al.* Distinct and complex bacterial profiles in human periodontitis and health revealed by 16S pyrosequencing. ISME J 6, 1176–1185, doi: 10.1038/ismej.2011.191 (2012). 22170420 PMC3358035

[b31] Li Y. *et al.* Phylogenetic and functional gene structure shifts of the oral microbiomes in periodontitis patients. ISME J 8, 1879–1891, doi: 10.1038/ismej.2014.28 (2014). 24671083 PMC4139721

[b32] Jiao Y., Hasegawa M. & Inohara N. The Role of Oral Pathobionts in Dysbiosis during Periodontitis Development. J. Dent. Res. 93, 539–546, doi: 10.1177/0022034514528212 (2014). 24646638 PMC4023464

[b33] Shi B. *et al.* Dynamic changes in the subgingival microbiome and their potential for diagnosis and prognosis of periodontitis. MBio 6, e01926–01914, doi: 10.1128/mBio.01926-14 (2015). 25691586 PMC4337560

[b34] Savage A., Eaton K. A., Moles D. R. & Needleman I. A systematic review of definitions of periodontitis and methods that have been used to identify this disease. J. Clin. Periodontol. 36, 458–467, doi: 10.1111/j.1600-051X.2009.01408.x (2009). 19508246

[b35] Querna J. C., Rossmann J. A. & Kerns D. G. Prevalence of periodontal disease in an active duty military population as indicated by an experimental periodontal index. Mil. Med. 159, 233–236 (1994). 8041472

[b36] Turesky S. S. What is the role of dental calculus in the etiology and progression of periodontal disease? J. Periodontol. 41, 285–286 (1970). 5267746

[b37] Huang S. *et al.* Preliminary characterization of the oral microbiota of Chinese adults with and without gingivitis. BMC Oral Health 11, 33, doi: 10.1186/1472-6831-11-33 (2011). 22152152 PMC3254127

[b38] Yang F. *et al.* Saliva microbiomes distinguish caries-active from healthy human populations. ISME J 6, 1–10, doi: DOI 10.1038/ismej.2011.71 (2012). 21716312 PMC3246229

[b39] Schloss P. D. *et al.* Introducing mothur: open-source, platform-independent, community-supported software for describing and comparing microbial communities. Appl. Environ. Microbiol. 75, 7537–7541, doi: 10.1128/AEM.01541-09 (2009). 19801464 PMC2786419

[b40] Griffen A. L. *et al.* CORE: a phylogenetically-curated 16S rDNA database of the core oral microbiome. PLoS One 6, e19051, doi: 10.1371/journal.pone.0019051 (2011). 21544197 PMC3081323

[b41] Breiman L. Random forests. Machine Learning 45, 5–32, doi: Doi 10.1023/A:1010933404324 (2001).

