# Supplementary material for: Microbiota-based Signature of Gingivitis Treatments: A Randomized Study
Source: Sci Rep. 2016 Apr 20;6:24705. doi: 10.1038/srep24705 (PMC4837389; doi:10.1038/srep24705)
Supplement: Supplementary Information [file srep24705-s1.pdf]

**Microbiota-based Signature of Gingivitis Treatments: A Randomized Study**

Shi Huang[a,f,g,1], Zhen Li[b,1], Tao He[c,1], Cunpei Bo[a], Jinlan Chang[d], Lin Li[d], Yanyan He[d], Jiquan Liu[e], Duane Charbonneau[c], Rui Li [e,2], Jian Xu[a,2]

[a] Single-Cell Center and Shandong Key Laboratory of Energy Genetics, Qingdao Institute of Bioenergy and Bioprocess Technology, Chinese Academy of Sciences, Qingdao, Shandong, 266101, China

[b] Department of Stomatology, Peking Union Medical College Hospital, Beijing, 100730, China

[c] Procter & Gamble Mason Business Center, Cincinnati, OH 45040, USA

[d] Procter & Gamble Innovation Center, Beijing 101312, China

[e] Procter & Gamble International Operations SA SG Branch, 138547, Singapore

[f] Sino-Danish Center for Education and Research, Beijing, 100190, China

[g] University of Chinese Academy of Sciences, Beijing, 100190, China

[1]These authors contributed equally to this work

[2]Correspondence: Tel: +86 532 8066 2651; fax: +86 532 8066 2654

E-mail address: xujian@qibebt.ac.cn or li.ru.5@pg.com

**Supplementary Information**

**Supplementary figures**

Figure S1

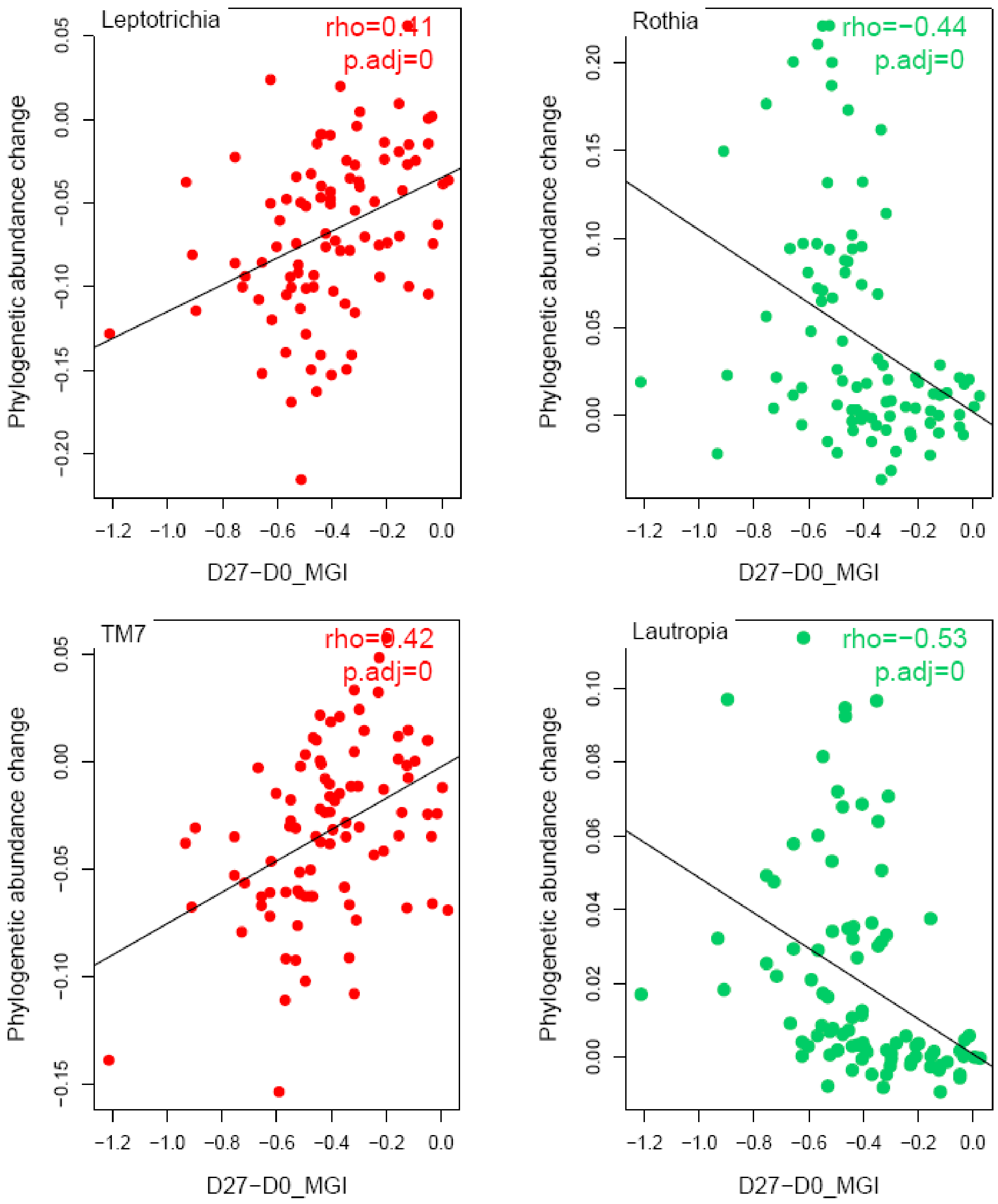


**Figure S1. Significant correlation between short-term changes in plaque bacteria from Baseline to Day 11, and mean MGI changes from Baseline to Day 27.**

Figure S2

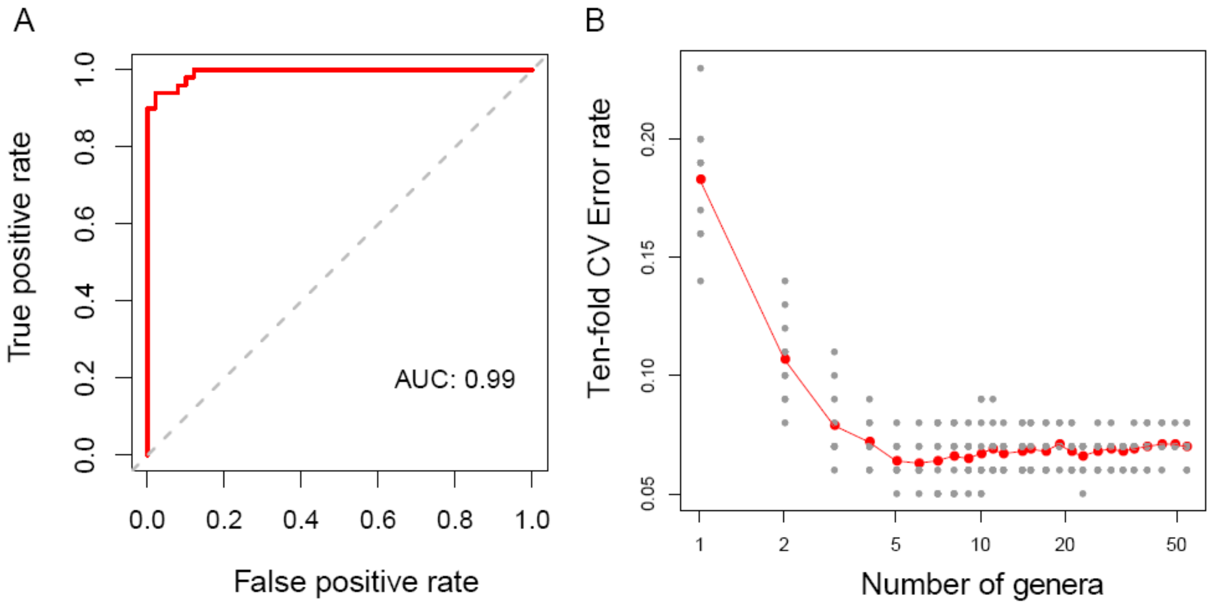


**Figure S2. Performance of the Random Forests model that was used to define “relative recovery of microbiota” during a given anti-gingivitis treatment.** Using the changes in plaque microbiota following dental scaling as a reference, the Random Forests model was trained using a data set of 50 subjects with gingivitis and post-dental-scaling healthy status from a previous study. (**A**) The discriminatory power of this model was calculated as the area under the ROC curve (AUC): 0.99. (**B**) Relationship between the number of variables and the model performance (ten-fold CV error rate).

Figure S3

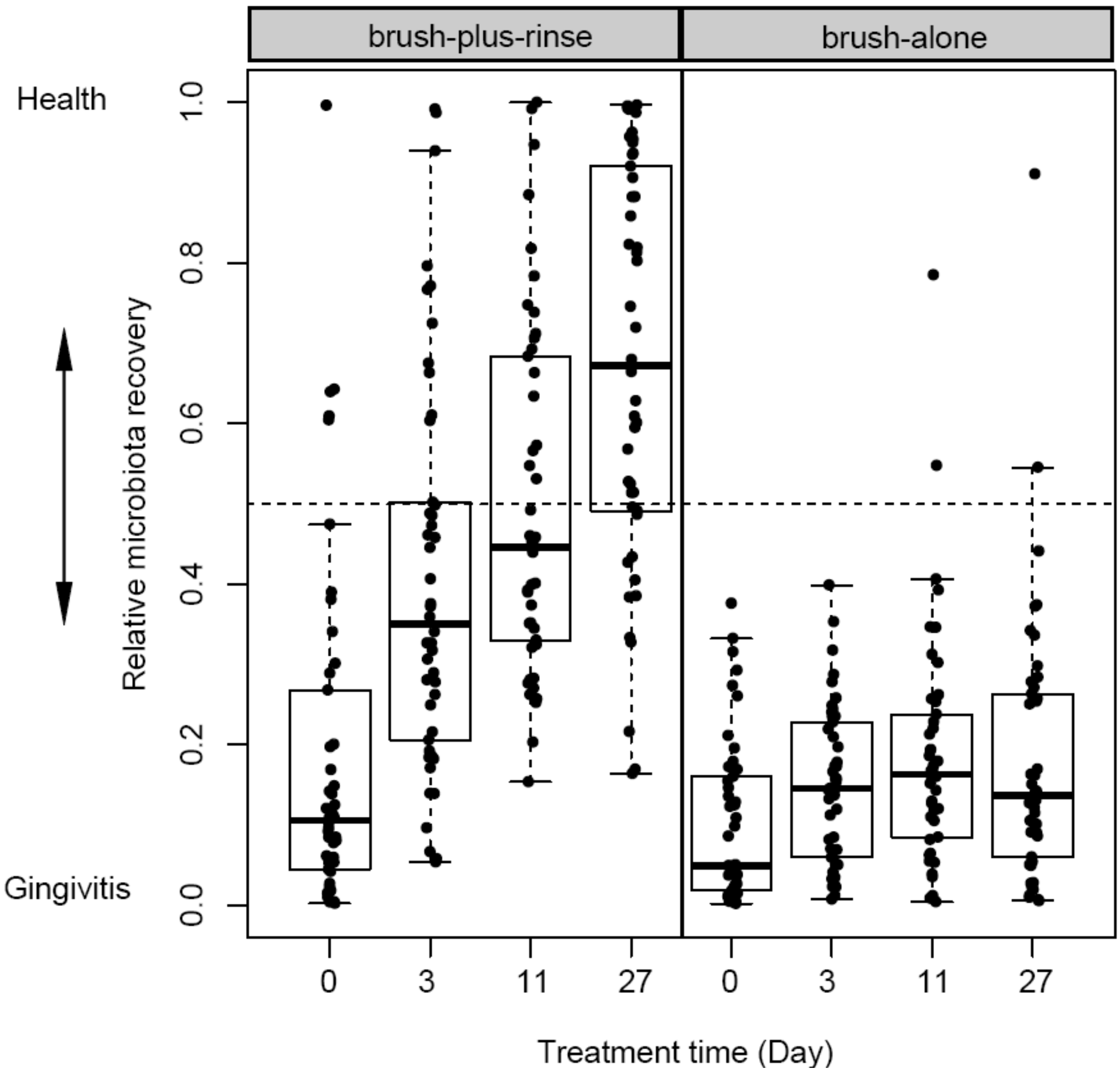


**Figure S3. Relative microbiota recovery defined by 15 genera identified in the MiG15 model.** Stratification of subjects in the brush-plus-rinse group and the brush-alone group, based on the top 15 genera previously noted as “MiG15”. MiG15, a microbial index of gingivitis, was calculated from the relative abundance of 15 bacterial genera (*Rothia, Haemophilus, Prevotella, Leptotrichia, Fusobacterium, Selenomonas, uncultured Lachnospiraceae, TM7, Tannerella, Peptococcus, Peptostreptococcus, Catonella, Treponema, Solobacterium and unclassified Bacteroidaceae*) [1].

## Reference


1 Huang, S. *et al.* Predictive modeling of gingivitis severity and susceptibility via oral microbiota. *ISME J* **8**, 1768-1780 (2014).
